# Automated Detection of Measurements and Their Descriptors in Radiology Reports Using a Hybrid Natural Language Processing Algorithm

**DOI:** 10.1007/s10278-019-00237-9

**Published:** 2019-06-20

**Authors:** Selen Bozkurt, Emel Alkim, Imon Banerjee, Daniel L. Rubin

**Affiliations:** 1 Department of Biomedical Data Science, Stanford University School of Medicine, Medical School Office Building (MSOB), Room X-335, MC 5464, 1265 Welch Road, Stanford, CA 94305-5479 USA; 2 Department of Radiology, Stanford University School of Medicine, Stanford, CA 94305 USA

**Keywords:** Measurement extraction, Natural language processing, Radiology report, Conditional random fields

## Abstract

Radiological measurements are reported in free text reports, and it is challenging to extract such measures for treatment planning such as lesion summarization and cancer response assessment. The purpose of this work is to develop and evaluate a natural language processing (NLP) pipeline that can extract measurements and their core descriptors, such as temporality, anatomical entity, imaging observation, RadLex descriptors, series number, image number, and segment from a wide variety of radiology reports (MR, CT, and mammogram). We created a hybrid NLP pipeline that integrates rule-based feature extraction modules and conditional random field (CRF) model for extraction of the measurements from the radiology reports and links them with clinically relevant features such as anatomical entities or imaging observations. The pipeline was trained on 1117 CT/MR reports, and performance of the system was evaluated on an independent set of 100 expert-annotated CT/MR reports and also tested on 25 mammography reports. The system detected 813 out of 806 measurements in the CT/MR reports; 784 were true positives, 29 were false positives, and 0 were false negatives. Similarly, from the mammography reports, 96% of the measurements with their modifiers were extracted correctly. Our approach could enable the development of computerized applications that can utilize summarized lesion measurements from radiology report of varying modalities and improve practice by tracking the same lesions along multiple radiologic encounters.

## Background

Radiology reports include a great variety of information about normal and abnormal structures in a free text format. Particularly for cancer patients, radiology reports describe measurements of cancer lesions and interval changes in their size are crucial indicators of response or resistance of cancer therapies. Measurements of lesion size (as well as organ size) are the predominant type of quantitative data recorded within the radiology reports. However, unlike other numerical, phenotypic evidence, such as lab values in ED notes, measurements are recorded as free text, which hampers extraction and utilization of such data by computer applications. Consequently, radiologists and clinicians need to ferret out lesion measurements from the radiology report for assessing changes in the tumor burden. Radiology reports typically capture lesion measurements, the anatomical locations, and spatial location—image and series number from where the measurements were taken. However, still, there are no widely adapted structured reporting standards for measurements in terms of its dimensions or descriptor terminology. In addition to measurement reporting, usage of different templates in general radiology reporting obstructs the automatic information extraction tasks.

Recent advancement in natural language processing (NLP) techniques could provide a fully automated solution for processing free text radiology reports to extract task-specific information, including measurements [1]. Yet, NLP techniques have been applied on radiological content either in the form of general-purpose systems or as targeted systems addressing one particular task [2–16]. Lesion measurement extraction and classification has been investigated in only a few studies [8, 13, 14, 17]. Sevenster et al. reported average F-measure 0.942 [12, 13] for classifying measurement descriptors; however, they did not link findings or anatomical locations to radiological measures. Similarly, Yim et al. extracted measurements from free text radiology reports as tumor characteristics but only focused on hepatocellular carcinoma patients [17].

Despite the increased use of the NLP approaches, radiology reports still introduce unique challenges for NLP, especially for granular tasks, such as determining anatomic relationships and temporal changes [18]. In addition to accurately extracting measurements, an NLP approach that can extract anatomic and spatial location of lesions and temporality of lesion measurements is also needed, which to our knowledge has not yet been undertaken. Moreover, no studies exist in literature which extract measurements as target concepts and links them with their descriptors of anatomical entities and imaging “slice (image) number” and “series number.” Recently, there are promising studies with advanced NLP methodologies in order to summarize radiology findings and generate “Impression” but so far measurements have not been extracted [19].

Therefore, the purpose of this work is to develop and evaluate an NLP engine that can extract measurements from narrative radiology reports with their core descriptors—“temporality,” “anatomical location and segment,” “imaging observation,” “scan-specific information”—“image number,” and “series number.”

## Methods

### Dataset

Under an Institutional Review Board (IRB)–approved protocol, we used a dataset of 980 CT and 237 MR reports from our institution for training and testing purposes. In terms of anatomic locations, our dataset consists of 782 chest/abdomen/pelvis, 52 head/neck, 157 lung/thorax, 44 pancreas, and 182 other types of reports. We randomly selected 100 radiology (26 MRI and 74 CT) reports from our data set to create our evaluation (test) set, and we used the remaining reports as our training set (1100) to train our conditional random field (CRF) model. In addition, we used a set of 25 mammography reports from our institution in order to evaluate the generalizability of our pipeline to other types of radiology reports.

### Proposed Pipeline

**Fig. 1:**
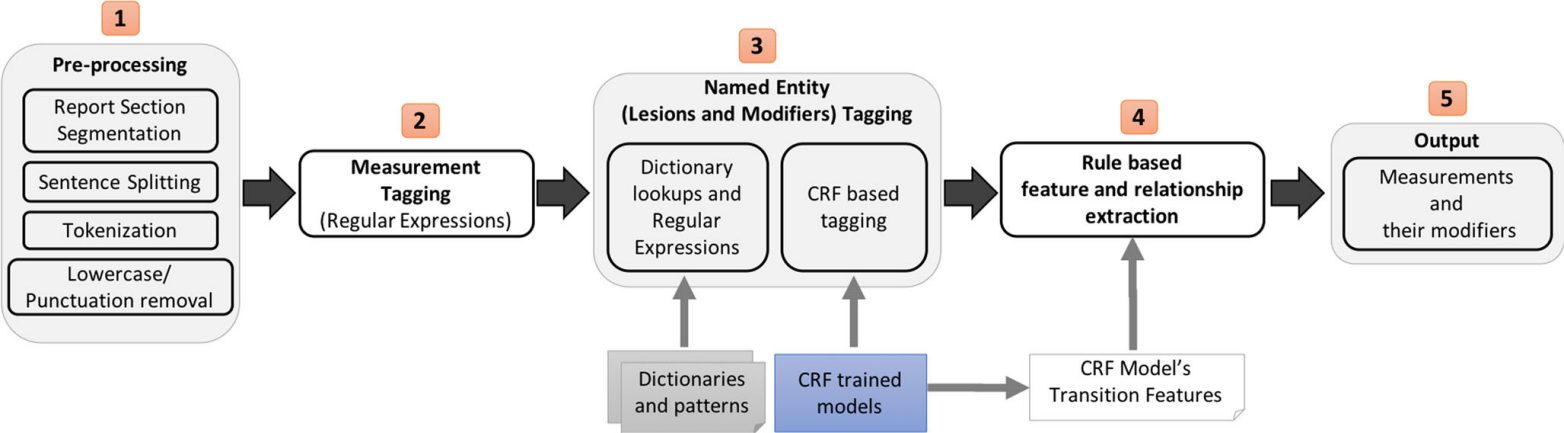
The proposed pipeline

In order to extract measurements from radiology reports with their descriptors, we created a rule-based NLP pipeline (Fig. 1) that includes automated named entity tagging using a CRF model. We also analyzed the label transition scores identified by the CRF model to explore relationships between descriptors. To compare the benefit of our approach over a commonly used approach, we created a baseline dictionary-based method that only uses terms in a dictionary as a knowledge source.

1. Pre-processing
  The reports were subjected to a boundary detection algorithm that recognizes sections and sentences in narrative radiology reports. The text was split into sections using regular expressions matched against a list of known section headers (commonly used in radiology) and segmented with respect to five sections of the report: “Comparison,” “Technique,” “Clinical History,” “Findings,” and “Conclusions.” Since lesion measurements are recorded in the Findings section, it was used in our pipeline as an input to extract descriptors of interest. All sections were decoded to ‘utf-8’ and were split into sentences using the Natural Language Toolkit (NLTK) [20] library in Python. In addition, all text was lowercased and punctuations were removed after measurements were tagged.
2. Measurement tagging
  In this phase, we aimed to tag measurements and their temporality which identifies whether a measurement is seen on the current scan or listed as a reference to a prior measurement. Measurements were tagged using several regular expression patterns and pre-defined rules. For measurement and temporality tagging, regex patterns defined by Sevenster et al. [14] were used with some modifications (Appendix 1). After measurement tagging, we only included the sentences that include measurements as the input for the following steps of the pipeline. Similarly, a complete textual description of all dimensions of a measurement in a sentence was targeted. In order to detect different measurements in the same sentence and tag temporality correctly as current or prior, we divided a sentence into sub-parts using the approach given as a pseudocode at Appendix 2. Basically, the sub-parts were created based on the number of measurements and their temporality. For example, the measurement sentence in Fig. 2 was divided into 2 parts as (1) current measurement part and (2) historical measurement part (Fig. 2). Similarly, other descriptors (image number series and series number on which the measurement is made, segment of the organ which measurements is made on) were extracted using several regular expression patterns and pre-defined rules. Regular expressions for image, series, and segment tagging were defined (Appendix 3).

3. Named entity (lesions and modifiers) tagging

**Fig. 2:**
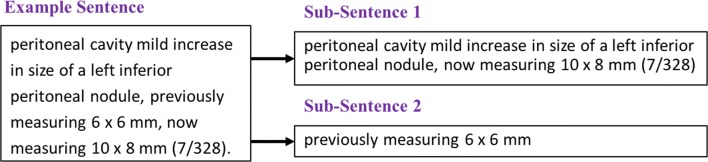
Example sentence to sub-sentence division to capture current and prior measures of the nodule
  We used a CRF method to adopt a more generalizable approach for named entity tagging since dictionary lookup might not able to capture all the lexical and linguistic variants of a medical term and radiology reports contain different writing styles depending upon the preference of radiologists and institutions. CRF is a probabilistic graphical model to discover patterns, given the context of a neighborhood, thus capture many correlated features of the inputs. CRF also helps to investigate the sequential relationships among the descriptors. The CRF model was trained to achieve automatic named entity tagging for anatomical entity, imaging observations, and RadLex descriptors (as RadLex sub-classes associated with the measurement). We also analyzed the label transition scores identified by the CRF model in order to explore and visualize relationships between descriptors. Label transition scores are the conditional probabilities of possible next states given the current state and the observation sequence [21]. As input feature to CRF, we used part of speech tagging and dictionary maps. We did not use any higher-level syntactic features like NP chunks, and we used the Python sklearn-crfsuite library (http://www.chokkan.org/software/crfsuite/) with its default model parameters.
4. Rule-based measurement descriptor extraction
  We mainly focused on 7 descriptors that characterize a measurement in radiology: (1) temporality, (2) anatomical entity, (3) imaging observation, (4) RadLex descriptor, (5) image number and (6) series number on which the measurement is reported, and (7) segment number of an organ. Output was recorded as frames, in which the measurement is the target entity and all other entities in the report are assumed to be related to the target entry as its descriptors. Thus, a secondary entity’s label encodes the type of the entity plus the type of relation with the target entity. Each measurement was represented as a single frame object containing the numeric measure of the lesion size and its descriptors as output from our pipeline (Fig. 3).

**Fig. 3:**
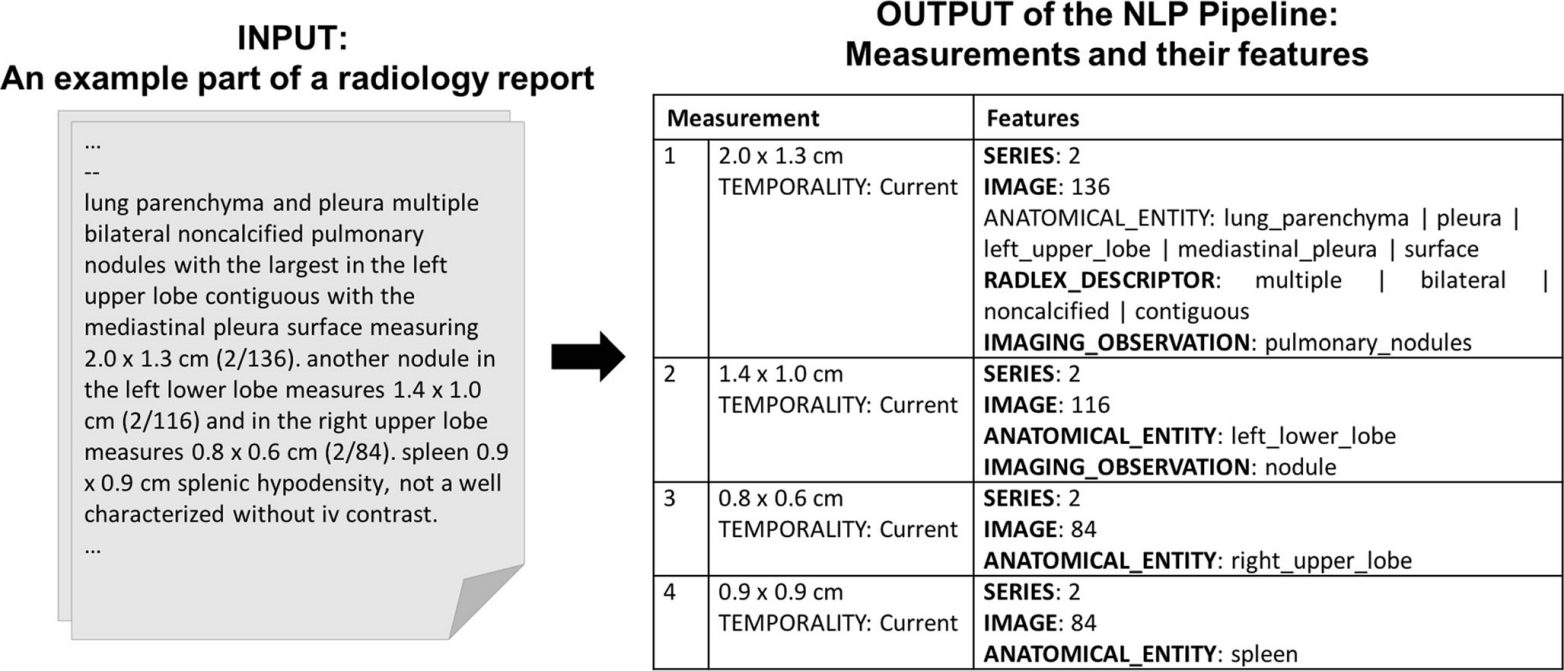
Example of the NLP system input and output where each measurement forms a frame object that summarizes all core properties

### Manual Annotation of the Reports

In order to evaluate the accuracy of the measurement extraction pipeline, we created a development (17 reports) and an evaluation set of randomly selected 100 radiology reports (26 MRI and 74 CT) and had them manually annotated by a domain expert. Reports were annotated to indicate measurements and their measurement descriptors (temporality, the image and series number, segment, anatomical entity, and imaging observation). Similarly, 25 mammography reports were also manually annotated by an expert.

In order to annotate the larger training set of 1100 reports for the CRF model, given its size, we used the “light annotation [22]” strategy in which we first generated the annotations for entities and relationships automatically via dictionary lookup and sentence boundaries; then, those annotations were manually corrected by experts to create the final set. This training set, being potentially lower quality than our evaluation set of annotations, were only used to train the CRF model and in creating rules of our pipeline.

### Statistical Evaluation

Using our evaluation set, we calculated precision, recall, and *F* scores for measurement extraction at the sentence level. The performance of extraction at report level was assessed as “no match,” “partial match,” and “full match.” If a measurement was extracted correctly with all of its descriptors, it was a “full match”, while, if even one descriptor was missed by the system, it was considered a “partial match”. “No match” occurred when the system failed to recognize any of the descriptors in addition to the measurement itself.

## Results

We compared the accuracy of our baseline and proposed pipeline with our manually annotated test set. The results of the proposed pipeline in terms of precision and recall are shown in Table 1.

**Table 1:** Evaluation of the NLP extraction pipelines from measure and its seven targeted descriptors

Information type	Baseline pipeline			The proposed pipeline		
	Precision	Recall	*F* score	Precision	Recall	*F* score
Measurement	96.43	100	98.18	96.43	100	98.18
Temporality (historical or not)	68.88	100	81.57	91.55	79.84	85.30
Anatomical entity	59.58	98.39	74.22	80.87	87.81	84.20
RadLex descriptor	57.02	99.75	72.56	67.35	83.19	74.44
Imaging observation	61.54	100	76.19	89.72	86.64	88.16
Segment	63.64	97.22	76.92	81.82	94.74	87.80
Image number	60.08	78.01	67.88	87.98	73.53	80.11
Series	63.71	78.67	70.41	89.77	72.34	80.12

The gold standard set of 100 reports contained a total of 806 reported measurements. Our system extracted 784 (97%) of them, and there were 29 (4%) false positives with no false negatives. Among 806 measurements, 258 of them were historical which referred to an earlier measurement and our system detected 206 (79.84%) of them correctly as prior measurements.

**Fig. 4:**
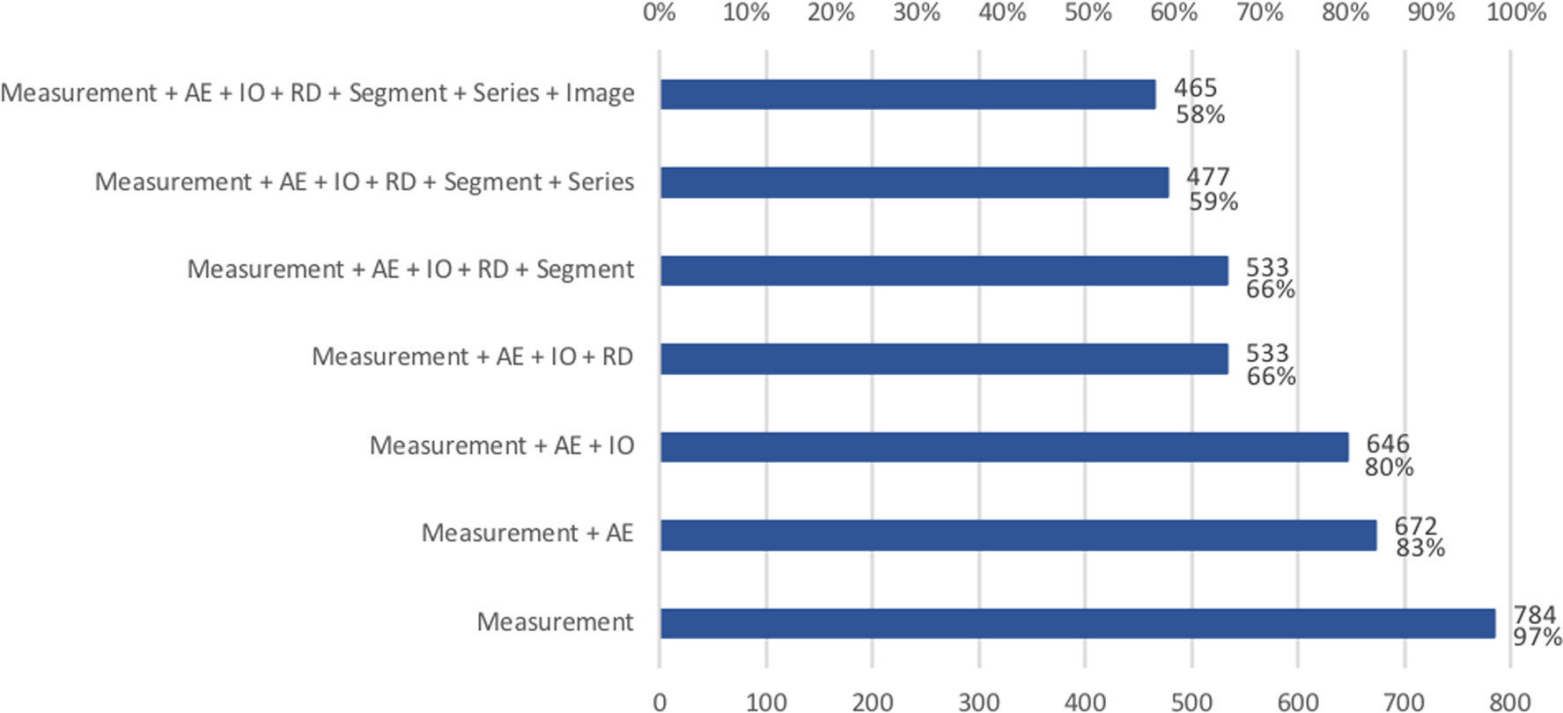
Results of the of the proposed pipeline in terms of partial and full matches. AE, anatomical entity; IO, imaging observation; RD, RadLex descriptors; Image, image number

For the goal of perfect-matched information frame extraction, we investigated the match percentages on the combination of descriptors. Figure 4 shows the final results for full and partial match cases with their frequencies and percentages based on the different descriptors of the measurements. The number of fully matched measurements was 465 (47.28%). Regarding partial matches, for example, 672 (83%) of the measurements were partial matches and they were matched at least with their anatomical entities correctly. It can be clearly seen in Fig. 4 that, when the number of descriptors related to a measurement increases, the number of full matches decreases.

**Fig. 5:**
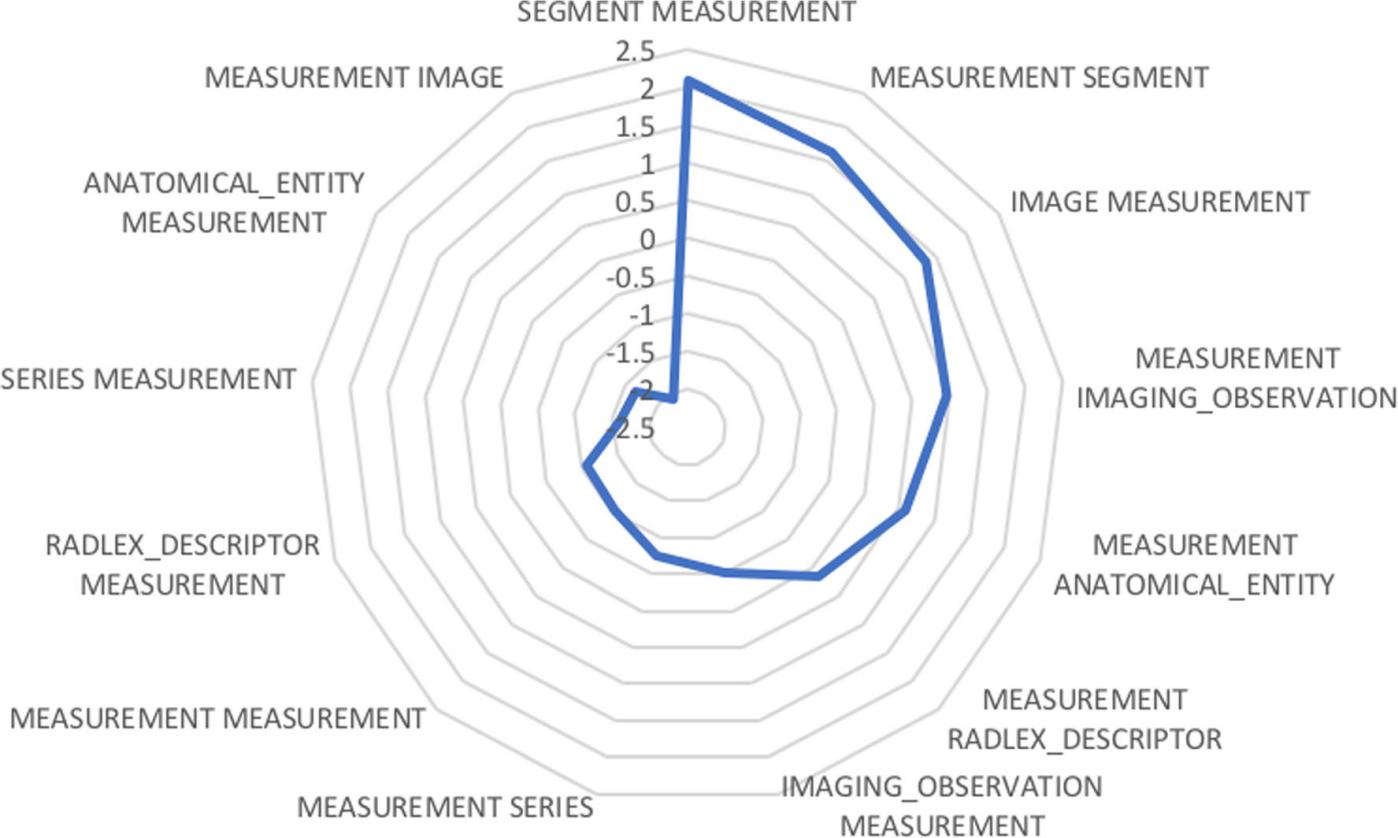
Label transition scores calculated by CRF

In order to explore and visualize relationships between descriptors using CRF-generated probability scores, all possible label transition scores among descriptors were illustrated in Fig. 5. We observed that a measurement is most likely to be followed by an imaging observation or anatomical entity but when it comes to segment and image number, it is difficult to detect the order of sequencing.

### Evaluation on Mammography Reports

Among 25 mammography reports included in our second evaluations set, 14 (56%) of them included multiple measurements in the same report. Among a total of 305 sentences in the dataset, 51 (17%) of those sentences included measurements and it corresponds to 51 unique measurements since none of the sentences had more than one measurement. Our measurement extraction pipeline extracted 49 (96%) of those measurements with their modifiers correctly (full match). The two cases that were extracted as false positive had an uncommon pattern as “increased in size (by 1 mm),” and our system extracted the measurement as “1 mm”.

## Discussion

In this paper, we describe an NLP system to extract measurements with their descriptors in a structured format from radiology reports. All of this information is necessary to track a lesion in a report over time, since the measurement itself is ambiguous, but the measurement in addition to its descriptors makes it sufficiently unique that it can be distinguished from other measurements in the report, enabling tracking of lesions. The recall and precision of our system for measurement extraction were 100% and 96.43%, respectively, which are reasonably good results for MR and CT reports. Among 784 (97%) correctly extracted measurements, only 465 (58%) measurements were extracted as fully matched with all of their related descriptors due to very diverse and unstructured referencing in text. On the other hand, in order to identify a measurement for any follow-up encounter, it is necessary to be able to distinguish them based on their all descriptors. As opposed to previous studies, in which measurements were defined as quantitative descriptions of other entities [23], in this study, we treated measurements as core concepts and defined other related entities as their descriptors. Errors we observed during the evaluation phase were primarily due to expressing several measurements in the same sentence with their previous measurements and insufficient descriptions of features for each measurement as in the example below.

### Example Sentence

“…reduced size of scattered enhancing nodules on series 11, for example 4 mm left frontal nodule (image 127, previously 7 mm), 4 mm right frontal nodule (image 124, previously 9 mm), 3 mm right frontal nodule (image 114; previously 7 mm), 2 mm lateral right frontal nodule (image 118, previously 5 mm), 2 mm left cerebellar nodule (image 53, previously 6 mm)….”

In this sentence, there are 10 different measurements (5 current and 5 prior) and their descriptors (imaging observations, RadLex descriptors, and image numbers). Our system finds each measurement correctly with its temporality, image/series numbers, and laterality. On the other hand, it only detected the “scattered|Radlex_Descriptor” and “enchancing|Imaging_Observation” for the first measurement since it is the closest and these modifiers are not reported in the other sub-sentences. Therefore, we calculated 9 of 10 cases as “partial match” and it decreased our system’s performance. These kinds of problems are due to a lack of description of each measurement separately, which might be solved by specific rules but it can also increase false positive cases.

One important goal of any information extraction task is to reveal the relations between concepts. However, relationship extraction is a granular task which includes several modifiers related to target measurement and requires detailed relationship labels generated for training a machine learning pipeline or purpose of rule development. Moreover, as a unique challenge of radiology report parsing, relationships between measurement and their characteristics are not obviously definable via adverbs, and relational and qualitative adjectives for all of the entities in our corpus except “Measure_of” anatomical entity. Therefore, we tried to learn entity sequencing using CRF models in order to provide some insights for associating modifiers with measurements via rules rather than directly using it as a relationship extraction model.

CRF is the most popular supervised machine learning algorithm for named entity tagging tasks. Being a statistical machine learning method, CRF analyzes the data to infer rules and patterns and uses sequence labeling to model relationships between neighbors [24, 25]. In this study, we trained a CRF model to label the named entities of interest automatically. We also aimed to mine relationships between measurement and their descriptors using the calculated transition probabilities of this model such as the following: a measurement is most likely to be followed by an imaging observation or anatomical entity, but we observed that it is difficult to decide about the order of sequencing for a given dataset with small training data set. Therefore, we only used the CRF model’s output for named entity tagging phase.

For the generalizability evaluation, we tested our system on 25 mammography reports and 96% of measurements were extracted correctly with their modifiers. Although the performance was very high, it should be noted that, in those reports, a single sentence does not include more than one measurement and our system performs best for sentences having only one measurement. On the other hand, this pattern would be common in mammography reports. As a future work, we are planning to evaluate the success of the pipeline on other modality reports.

The main limitation of this study was the small dataset from one single institution; in our future experiments, we plan to increase the training and test size with reports from multiple different institutions, hence, increasing the generalizability of our system. Similarly, due to limited resource, we performed a “light annotation [22]” of the training set of (1100 reports) for CRF model by a single expert. On the other hand, the annotation of the test set was a completely manual effort which we think is a valuable resource that we will use in future work for developing appropriate lesion tracking models. In the future, we also intend to adopt attention-based convolutional neural network model for extracting relations between the entities. It should be noted that all these methodologies require larger training sets and manual annotation of the training data is a very labor-intensive task.

Extracting measurements and their descriptors as a structured summary of the lesions from unstructured radiology reports might be quite valuable for lesion tracking purposes. That information might be used to disambiguate the lesions across studies to identify the baseline and follow-up measurements of the same lesion. For example, if a lesion in the fifth segment of the liver is identified in the baseline study and then, it is identified again in the follow-up study, the anatomical entity and the segment number can be used to associate the measurements as the measurement of the same lesion. Moreover, the historical references can be used to bind a measurement to the measurement of the same lesion in a prior study. This can help in generating automatic lesion tracking and tumor burden reports. In addition, another impact of automated text annotation, such as in our work, is large-scale data labeling to train models that automate image interpretation.

## Conclusion

Notwithstanding the foregoing limitations and challenges, we believe there is potential for clinical utility of our approach to improve radiologist practice by enabling automatic measurement extraction and summarization from radiology reports. With further testing, the system may ultimately help to improve radiology practice by enabling automated lesion summary, facilitate the assessment of changes in tumor burden, and improve the quality of patient care.

## Measurement and temporality extraction

Measurement and auxiliary regular expressions are used same as they are defined by Sevenster et al. [14]. m specifies the measurement regular expression to match; using other regular expressions listed under Measurement. x specifies the numerical part of the measurement whereas cm specifies the unit. Auxiliary regular expressions are helper regular expressions that are used in prior regular expressions to match temporality.

Sevenster et al. [14] defined regular expressions for both current and prior. However, in this study, we used only prior regular expressions and tagged every measurement that is not matched by prior regular expressions as current. p5 was removed from the regular expressions for temporality (prior) regular expressions, and five new ones were defined (p0, p12, p13, p14, p15).

## Pseudocode

The pseudocode listed below specifies three significant functionalities of the pipeline. The main flow of the tagging process is listed in *tag*_*reports*. Two methods that are used during tagging processes *split_according_to_temporality* and *split_for_measurement* are also listed to clarify the algorithm behind splitting which plays a significant role in extracting the relationships between modifiers and measurements.

## Series number, image number, and segment extraction

Series number, image number, and segment are extracted using the regular expressions below. Segment regular expression is defined to also handle roman numerals.
